# Cardiovascular protection beyond glucose lowering: GLP-1 receptor agonists and dual GIP/GLP-1 receptor agonism in contemporary cardiology

**DOI:** 10.3389/fcvm.2026.1926629

**Published:** 2026-09-16

**Authors:** Mingzhe Li

**Affiliations:** Department of Cardiology, Siping City Central People’s Hospital, Siping, China

**Keywords:** cardiovascular disease, GLP-1 receptor agonists, HFPEF, MACE, obesity, tirzepatide

## Abstract

Glucagon-like peptide-1 receptor agonists (GLP-1RAs) and the dual glucose-dependent insulinotropic polypeptide (GIP)/GLP-1 receptor agonist tirzepatide have expanded from glucose-lowering therapy into cardiovascular medicine. In type 2 diabetes, several long-acting GLP-1RAs reduce major adverse cardiovascular events (MACE). SELECT extended this benefit to adults with established cardiovascular disease and overweight or obesity without diabetes, while SOUL demonstrated MACE reduction with oral semaglutide. SURPASS-CVOT established cardiovascular noninferiority of tirzepatide to dulaglutide but did not demonstrate superiority. Obesity-related heart failure with preserved ejection fraction (HFpEF) has emerged as a particularly responsive phenotype: semaglutide improves symptoms and physical function, whereas tirzepatide also reduces worsening heart failure events. FLOW further supports kidney and cardiovascular protection with semaglutide in type 2 diabetes and chronic kidney disease. Randomised human studies support effects on adiposity, haemodynamic burden, inflammation and cardiorenal outcomes, whereas endothelial, coronary microvascular and direct myocardial mechanisms remain less clinically validated. This Review integrates the current evidence, distinguishes randomised outcomes from observational and mechanistic signals, and provides a practical phenotype-directed treatment algorithm incorporating atherosclerotic cardiovascular disease, obesity, type 2 diabetes, chronic kidney disease, HFpEF, sodium–glucose cotransporter 2 inhibitor use and patient-specific treatment constraints. Current practice should be guided by indication-specific outcome evidence rather than weight-loss magnitude or indirect cross-trial comparisons.

## 1 Introduction

Cardiovascular disease increasingly reflects the convergence of obesity, type 2 diabetes, chronic kidney disease, ectopic adiposity and systemic inflammation, which contribute to residual risk despite conventional preventive treatment. GLP-1 receptor agonists (GLP-1RAs) are relevant to contemporary cardiology because they modify several components of this cardiometabolic substrate rather than glycaemia alone.

Early cardiovascular outcome trials primarily established safety, whereas LEADER, SUSTAIN-6, Harmony Outcomes, REWIND and AMPLITUDE-O subsequently demonstrated reductions in three-point major adverse cardiovascular events (MACE) with several long-acting GLP-1RAs (1–6). SELECT extended cardiovascular benefit to adults with established cardiovascular disease and overweight or obesity without diabetes (7–9). STEP-HFpEF and SUMMIT identified obesity-related heart failure with preserved ejection fraction (HFpEF) as an incretin-responsive phenotype (10–18), while SOUL and SURPASS-CVOT updated the evidence for oral semaglutide and tirzepatide in high-risk type 2 diabetes (19–23).

GLP-1RAs improve glycaemia and body weight through glucose-dependent insulin secretion, suppression of inappropriate glucagon release, delayed gastric emptying and reduced appetite (24). Tirzepatide activates both GIP and GLP-1 receptors and produces greater average reductions in HbA1c and body weight than many selective GLP-1RAs. However, metabolic potency should not be assumed to confer superior cardiovascular protection, because cardiovascular outcomes depend on the agent, population, comparator, follow-up and endpoint studied.

Direct myocardial GLP-1 receptor signalling remains uncertain in humans, and cardiovascular benefit is more plausibly explained by convergent metabolic, vascular, haemodynamic and cardiorenal effects. Direct randomised outcome trials form the primary basis for clinical conclusions and treatment recommendations in this Review. Meta-analyses and prespecified randomised subgroup analyses are used to assess consistency and treatment-effect heterogeneity, whereas observational and real-world studies are considered supportive evidence for transportability, comparative effectiveness, safety and implementation. Such studies are not interpreted as substitutes for randomised evidence because residual confounding and treatment-selection bias cannot be fully excluded. Mechanistic and preclinical findings are discussed separately as sources of biological plausibility.

## 2 Atherosclerotic cardiovascular disease and major cardiovascular events

### 2.1 Lessons from the diabetes cardiovascular outcome trials

The diabetes cardiovascular outcome trials established that MACE reduction is not uniform across all glucose-lowering agents or all incretin drugs. In LEADER, liraglutide reduced three-point MACE by 13% relative to placebo; SUSTAIN-6 reported a 26% relative reduction with injectable semaglutide; Harmony Outcomes showed a 22% reduction with albiglutide; REWIND demonstrated a smaller but significant 12% reduction with dulaglutide in a population with a lower proportion of established cardiovascular disease; and AMPLITUDE-O reported a 27% reduction with efpeglenatide (1–4, 6). PIONEER 6 established cardiovascular safety for oral semaglutide but was not powered to demonstrate superiority (5).

Cardiovascular efficacy has not been demonstrated uniformly across individual GLP-1RAs. ELIXA and EXSCEL established cardiovascular safety for lixisenatide and once-weekly exenatide, respectively, but neither demonstrated MACE superiority. Differences in molecular structure, duration of receptor activation, adherence, baseline risk, follow-up and statistical power may contribute to this heterogeneity. Meta-analyses support an overall class-level cardiovascular signal (25, 26), but agent-specific treatment recommendations should remain anchored to direct randomized outcome evidence.

Importantly, cardiovascular efficacy has not been demonstrated uniformly across all GLP-1 receptor agonists. ELIXA showed that lixisenatide was cardiovascularly safe but did not reduce major cardiovascular events after acute coronary syndrome, whereas EXSCEL demonstrated noninferiority, but not superiority, of once-weekly exenatide for three-point MACE. PIONEER 6 similarly established cardiovascular safety for oral semaglutide but was not powered to demonstrate superiority (5). By contrast, liraglutide, subcutaneous semaglutide, albiglutide, dulaglutide and efpeglenatide achieved significant MACE reductions in their respective cardiovascular outcome trials (1–4, 6).

The reasons for this heterogeneity remain uncertain. Molecular structure, duration of receptor activation, trial population, baseline cardiovascular risk, adherence, follow-up duration and statistical power may all contribute, but no single factor fully explains the differences. Meta-analyses support an overall class-level reduction in cardiovascular events (26–28); however, a pooled class estimate should not be used to assign proven cardiovascular efficacy to an individual agent without agent-specific randomised outcome evidence.

Cardiovascular protection cannot be attributed to a single mediator. Changes in HbA1c, body weight, blood pressure, albuminuria and inflammation explain only part of the observed benefit, and effect estimates vary across agents and trial populations (26–28). Superiority for MACE has been demonstrated with liraglutide, subcutaneous semaglutide, albiglutide, dulaglutide and efpeglenatide, whereas lixisenatide and exenatide were neutral. Overall, GLP-1RAs are most consistently associated with atherosclerotic outcomes; reductions in heart failure hospitalisation are smaller and less consistent than with sodium–glucose cotransporter 2 (SGLT2) inhibitors (25, 26, 28).

Accordingly, the evidence supports a clinically meaningful class-level signal, but treatment recommendations should remain anchored to the outcome evidence available for the individual agent and the population studied. GLP-1RAs should be used as an adjunct to, not a replacement for, standard secondary prevention. Lipid-lowering therapy, antithrombotic treatment, blood-pressure control, smoking cessation and evidence-based heart-failure or kidney therapies remain foundational (29–32).

### 2.2 Semaglutide and secondary prevention beyond diabetes

SELECT was a conceptual turning point because it enrolled 17,604 adults with established cardiovascular disease and a body mass index of at least 27 kg/m^2^, but without diabetes. Semaglutide 2.4 mg reduced cardiovascular death, nonfatal myocardial infarction or nonfatal stroke by 20% relative to placebo (hazard ratio 0.80) over a mean follow-up of approximately 40 months (7). SELECT established obesity as a modifiable cardiovascular treatment target independent of diabetes status and provided direct secondary-prevention evidence for semaglutide in the population studied. Prespecified analyses support consistency across baseline glycaemic categories and in participants with prevalent heart failure (8, 9). SCORE and SCORE–Clinical ASCVD provide supportive information on transportability but do not extend or supersede the randomised efficacy evidence (33, 34).

Prespecified SELECT analyses strengthen, but do not radically change, the primary interpretation. Benefit was broadly consistent across baseline glycated hemoglobin categories and was not explained by changes in HbA1c, supporting a mechanism beyond glucose lowering (8). Participants with prevalent heart failure also appeared to derive cardiovascular benefit, although SELECT was not designed as a dedicated heart failure efficacy trial and did not replace the phenotype-specific evidence from STEP-HFpEF or SUMMIT (9). The trial population was predominantly composed of patients with established atherosclerotic disease, so extrapolation to primary prevention or to lean patients remains uncertain.

The applicability of SELECT is strongest for secondary prevention in adults with established atherosclerotic cardiovascular disease (ASCVD) and overweight or obesity. Extrapolation to low-risk primary prevention or lean populations remains unsupported. Coronary artery calcium and other measures of plaque burden may refine estimates of absolute benefit, but they have not been shown to predict a differential biological response to semaglutide (35–37). Whether incretin-based therapy prevents first cardiovascular events in lower-risk populations with obesity remains uncertain. Future research should determine which primary-prevention populations have sufficient absolute cardiovascular risk to achieve clinically meaningful benefit and should distinguish prevention of metabolic risk factors from prevention of major cardiovascular events.

Real-world analyses provide supportive information on the transportability of SELECT but do not independently establish treatment efficacy. SCORE and SCORE–Clinical ASCVD reported lower cardiovascular event rates among semaglutide 2.4 mg initiators than among non-initiators in routine care (33, 34). However, treatment initiation may be associated with socioeconomic status, health behaviour, clinical follow-up, medication adherence and use of other preventive therapies. These findings are therefore consistent with, but do not extend or supersede, the randomised evidence from SELECT.

### 2.3 Semaglutide and tirzepatide: cardiovascular evidence and limits of comparison

PIONEER 6 established the cardiovascular safety of oral semaglutide but was not powered to demonstrate superiority (5). SOUL subsequently enrolled patients with type 2 diabetes and established atherosclerotic cardiovascular disease, chronic kidney disease or both, and demonstrated a 14% relative reduction in three-point MACE with oral semaglutide compared with placebo (19). The cardiovascular benefit was consistent in participants receiving or not receiving an SGLT2 inhibitor, without a major safety signal from combined treatment (20). A secondary analysis also found no evidence of harm in patients with heart failure and suggested favourable effects on heart failure-related outcomes (38). These findings extend proven cardiovascular efficacy to an oral GLP-1RA formulation, although fasting administration requirements and the need for daily adherence may limit convenience for some patients.

SURPASS-CVOT provided a more stringent active-comparator assessment of tirzepatide. More than 13,000 patients with type 2 diabetes and established atherosclerotic cardiovascular disease were randomly assigned to tirzepatide or dulaglutide, a GLP-1RA with established cardiovascular benefit. Tirzepatide was noninferior to dulaglutide for cardiovascular death, myocardial infarction or stroke, with a hazard ratio of 0.92 and a 95.3% confidence interval of 0.83–1.01, but did not meet the prespecified criterion for superiority (21). Tirzepatide produced greater reductions in body weight and HbA1c, demonstrating that greater metabolic efficacy does not necessarily translate into statistically superior MACE reduction over the duration of a cardiovascular outcome trial.

A subsequent *post hoc* analysis reported favourable effects of tirzepatide on broader cardiorenal composite outcomes compared with dulaglutide, but these exploratory findings should remain distinct from the prespecified primary endpoint (22). Earlier pooled trial data also supported the cardiovascular safety of tirzepatide, but were not a substitute for the dedicated active-comparator outcome trial (23). The appropriate conclusion is therefore that tirzepatide has proven cardiovascular noninferiority to an evidence-based GLP-1RA in high-risk type 2 diabetes, rather than demonstrated cardiovascular superiority over the GLP-1RA class.

No randomised cardiovascular outcome trial has directly compared tirzepatide with semaglutide. SURPASS-CVOT compared tirzepatide with dulaglutide and demonstrated noninferiority, but not superiority, for the primary three-point MACE endpoint (21). Its findings therefore cannot establish superiority over semaglutide or over the GLP-1RA class as a whole. The greater reductions in body weight and HbA1c achieved with tirzepatide should likewise not be interpreted as evidence of greater atherosclerotic cardiovascular protection.

Indirect comparisons across SELECT, SOUL, SURPASS-CVOT, STEP-HFpEF and SUMMIT are limited by substantial differences in diabetes status, cardiovascular phenotype, baseline risk, active vs. placebo comparators, drug dose, follow-up duration and endpoint definition. In particular, semaglutide and tirzepatide have not been compared directly in either a cardiovascular outcome trial or an obesity-related HFpEF trial. Numerical differences between studies therefore cannot be interpreted as comparative treatment effects.

Observational comparisons have also produced inconsistent results. One international cohort suggested lower cardiovascular event rates with tirzepatide, whereas a subsequent trial-emulation analysis reported broadly similar cardiovascular effectiveness after adjustment (39, 40). Although such studies provide information on routine-practice use, residual confounding, differences in dose attainment, treatment access and patient selection remain unavoidable. Semaglutide and tirzepatide should therefore be described according to their indication-specific evidence portfolios rather than ranked globally as superior or inferior.

Semaglutide and tirzepatide currently have distinct, partially overlapping evidence portfolios. Semaglutide has direct evidence for secondary prevention in overweight or obesity without diabetes and replicated symptomatic and functional efficacy in obesity-related HFpEF, whereas tirzepatide has demonstrated reduction of worsening heart failure events in SUMMIT and cardiovascular noninferiority to dulaglutide in SURPASS-CVOT. These differences do not establish the overall cardiovascular superiority of either agent.

The designs, populations, primary cardiovascular endpoints and principal findings of the pivotal cardiovascular outcome trials of GLP-1 receptor agonists and tirzepatide are summarised in Table 1.

**Table 1:** Pivotal cardiovascular outcome trials of GLP-1 receptor agonists and dual GIP/GLP-1 receptor agonists.

Trial	Agent	Population	*N*	Comparator	Endpoint	Result	Interpretation
ELIXA	Lixisenatide	T2D + recent ACS	6,068	Placebo	CV death/MI/stroke/UA	HR 1.02	CV safety demonstrated; no MACE superiority
LEADER	Liraglutide	T2D + CVD/high risk	9,340	Placebo	3-point MACE	HR 0.87	↓ MACE
SUSTAIN-6	SC semaglutide	T2D + CVD/CKD/high risk	3,297	Placebo	3-point MACE	HR 0.74	↓ MACE (noninferiority met)
EXSCEL	Exenatide QW	T2D ± CVD	14,752	Placebo	3-point MACE	HR 0.91	CV safety demonstrated; MACE superiority not achieved
Harmony	Albiglutide	T2D + CVD	9,463	Placebo	3-point MACE	HR 0.78	↓ MACE
REWIND	Dulaglutide	T2D + CVD/risk	9,901	Placebo	3-point MACE	HR 0.88	↓ MACE
PIONEER 6	Oral semaglutide	T2D + CVD/CKD/high risk	3,183	Placebo	3-point MACE	HR 0.79	CV safety demonstrated; not powered for superiority
AMPLITUDE-O	Efpeglenatide	T2D + CVD/CKD	4,076	Placebo	3-point MACE	HR 0.73	↓ MACE
SELECT	Semaglutide 2.4 mg	CVD + obesity (no T2D)	17,604	Placebo	3-point MACE	HR 0.80	↓ MACE (non-diabetic)
SOUL	Oral semaglutide	T2D + ASCVD/CKD	9,650	Placebo	3-point MACE	HR 0.86	↓ MACE
SURPASS-CVOT	Tirzepatide	T2D + ASCVD	13,299	Dulaglutide	3-point MACE	HR 0.92	Noninferior to dulaglutide; superiority not demonstrated

Between-trial differences should not be interpreted as direct evidence of superiority or inferiority among agents because study populations, comparators, follow-up durations and endpoint definitions differed.

Abbreviations: ACS, acute coronary syndrome; ASCVD, atherosclerotic cardiovascular disease; CI, confidence interval; CKD, chronic kidney disease; CVD, cardiovascular disease; GIP, glucose-dependent insulinotropic polypeptide; GLP-1, glucagon-like peptide-1; HR, hazard ratio; MACE, major adverse cardiovascular events; MI, myocardial infarction; T2D, type 2 diabetes.

Semaglutide and tirzepatide should therefore be compared across specific evidence domains rather than ranked according to weight loss or numerical results from separate trials. Table 2 provides an integrated comparison of their pharmacological profiles, evidence-supported clinical settings, cardiovascular outcome data, heart-failure evidence, kidney evidence, real-world findings, principal strengths, limitations and remaining uncertainties. The comparison distinguishes direct randomised evidence from *post hoc* and observational findings and should not be interpreted as evidence of overall superiority of either agent.

**Table 2:** Integrated comparison of semaglutide and tirzepatide in cardiovascular and cardiorenal medicine.

Domain	Semaglutide	Tirzepatide
Pharmacological strategy	Selective GLP-1 receptor agonist	Dual GIP/GLP-1 receptor agonist
ASCVD outcome evidence	SUSTAIN-6, SELECT and SOUL	SURPASS-CVOT
HFpEF evidence	STEP-HFpEF programme	SUMMIT
Kidney evidence	FLOW	Post hoc kidney signals; dedicated outcome evidence less mature
Supportive real-world evidence	SCORE studies and comparative cohorts	Comparative cohorts and trial-emulation studies
Principal strengths	Broader direct evidence across several phenotypes	Greater average metabolic potency and worsening-HF event evidence
Principal limitations	No direct comparison with tirzepatide; hard HF outcome evidence limited	MACE superiority not demonstrated; dedicated kidney outcome evidence lacking
Remaining uncertainties	Comparative effectiveness and broader HF populations	Whether greater metabolic potency translates into greater cardiovascular protection
Overall evidence boundary	No direct randomized evidence of overall superiority between the two agents	No direct randomized evidence of overall superiority between the two agents

The table summarizes evidence-supported clinical settings and does not represent jurisdiction-specific regulatory labelling. Comparisons across separate trials should not be interpreted as direct comparative treatment effects because study populations, comparators, follow-up durations and endpoint definitions differed. Randomised outcome trials form the primary basis for efficacy conclusions. Observational and real-world findings are included to assess transportability and routine-practice effectiveness and should not be interpreted as substitutes for randomised comparisons.

## 3 Heart failure: obesity-related HFpEF as an incretin-responsive phenotype

### 3.1 Why obesity-related HFpEF is biologically distinct

HFpEF is a syndrome rather than a single disease. Obesity-related HFpEF is characterized by plasma-volume expansion, increased filling pressures, systemic inflammation, impaired skeletal-muscle oxygen extraction, sleep apnoea, renal congestion and excess epicardial or paracardiac adipose tissue. These abnormalities are not secondary cosmetic features; they contribute directly to exercise intolerance, atrial remodelling and haemodynamic instability. A therapy that reduces adiposity while improving inflammatory and renal-metabolic stress is therefore especially plausible in this phenotype.

This phenotype-based concept also explains why results should not be generalized to all patients with preserved ejection fraction. Amyloidosis, significant valvular disease, hypertrophic cardiomyopathy, pulmonary vascular disease and lean hypertensive HFpEF have different dominant mechanisms. Incretin therapy may still be appropriate for diabetes or obesity in such patients, but direct HFpEF disease modification has not been established outside an obesity-enriched population.

Obesity-related HFpEF emerges from the interaction of haemodynamic loading and metabolic inflammation. Expanded blood volume and renal sodium retention increase cardiac filling pressure; visceral adiposity raises intra-abdominal and intrathoracic pressure; and arterial stiffness increases ventricular afterload. Simultaneously, adipose-derived cytokines and oxidative stress impair endothelial nitric-oxide signalling and coronary microvascular reserve. The result is reduced diastolic compliance, limited systolic reserve during exercise and abnormal peripheral oxygen extraction even when resting ejection fraction appears normal (41–43).

The skeletal muscle and pulmonary circulation are integral to this syndrome. Patients often have reduced capillary density, mitochondrial dysfunction and impaired oxygen utilisation in locomotor muscle. Sleep-disordered breathing and pulmonary vascular dysfunction further amplify exertional dyspnoea. These mechanisms help explain why a therapy that reduces weight, improves mobility, lowers inflammation and modifies volume distribution can produce a large improvement in Kansas City Cardiomyopathy Questionnaire (KCCQ) without a dramatic change in resting ejection fraction. Treatment therefore targets the broader cardiometabolic phenotype rather than a single echocardiographic variable.

Diabetes and chronic kidney disease may intensify obesity-related HFpEF through metabolic inflexibility, microvascular dysfunction and renal congestion (42). Diagnosis should confirm that obesity is a major disease driver and exclude mimics such as amyloidosis, major valvular disease, hypertrophic cardiomyopathy, constriction and severe lung disease (41–43). Incretin therapy should then be integrated with established HFpEF care: SGLT2 inhibitors and diuretics remain foundational when indicated, whereas semaglutide or tirzepatide are most relevant when obesity is a major contributor to symptoms and haemodynamic burden.

### 3.2 Semaglutide: symptoms, function and treatment burden

STEP-HFpEF randomised 529 patients with HFpEF and obesity to semaglutide 2.4 mg or placebo for 52 weeks. Semaglutide produced clinically meaningful improvements in the Kansas City Cardiomyopathy Questionnaire clinical summary score, reduced body weight, increased six-minute walk distance and lowered C-reactive protein concentrations (10). STEP-HFpEF DM reproduced the symptomatic and functional benefits in patients with type 2 diabetes, although the magnitude of weight loss was smaller than in participants without diabetes (11). A pooled analysis of the two trials confirmed consistent improvements in health status, physical function and inflammatory burden across the STEP-HFpEF programme (12).

Prespecified and secondary analyses showed that the benefits were broadly consistent across obesity classes and in both women and men (13, 14). Participants with atrial fibrillation (AF) also experienced improvements in symptoms and function (44). Greater achieved weight loss was associated with larger improvements in health status, supporting adiposity reduction as an important mediator. However, this relationship cannot establish causality because achieved weight loss is a post-randomisation variable and may also reflect treatment tolerance, adherence and other favourable patient characteristics.

Semaglutide additionally reduced loop-diuretic requirements and the likelihood of diuretic initiation or intensification (15). This finding is compatible with reduced congestion and haemodynamic burden, but may also reflect lower body mass, altered sodium intake or improved renal physiology. Because diuretic use was not the primary randomised endpoint, it should be considered supportive rather than definitive evidence of a direct natriuretic or myocardial effect.

The principal strength of the STEP-HFpEF programme is the consistency of its patient-centred findings across symptoms, walking capacity, body weight and inflammatory markers. Its principal limitation is that the trials were not powered to determine effects on cardiovascular death or heart failure hospitalisation. Semaglutide should therefore be described as an evidence-based treatment for symptoms, physical limitation and excess adiposity in obesity-related HFpEF, rather than as a therapy with independently proven mortality benefit in this population.

Clinical follow-up should extend beyond body weight. Symptoms, orthostatic blood pressure, oedema, jugular venous pressure, kidney function, electrolytes, diuretic dose, appetite, nutritional status and physical performance should be reassessed during dose escalation. Improvement should ideally be multidimensional, with better health status and mobility, less congestion and preservation of muscle function. Conversely, weight loss accompanied by weakness, symptomatic hypotension or kidney deterioration should prompt reassessment of dose escalation, oral intake and concomitant diuretic or antihypertensive therapy.

### 3.3 Tirzepatide: clinical outcomes and structural remodeling

SUMMIT extended the evidence in obesity-related HFpEF from symptom improvement to adjudicated heart failure events. Among 731 patients with HFpEF and obesity, tirzepatide reduced the composite of cardiovascular death or worsening heart failure, with a hazard ratio of 0.62, and also improved health status and walking distance (16). The primary composite benefit was driven predominantly by fewer worsening heart failure events. Cardiovascular deaths were uncommon, and the trial was not sufficiently powered to establish an independent mortality benefit. The most accurate interpretation is therefore that tirzepatide reduces worsening heart failure events and improves patient-centred outcomes in obesity-related HFpEF, rather than that it has demonstrated a survival advantage.

Human imaging and biomarker substudies provide supportive, but not causal, mechanistic evidence for the clinical findings. Cardiac magnetic resonance imaging demonstrated reductions in left-ventricular mass and paracardiac adipose tissue, while a secondary analysis reported improvements in indices of circulatory overload, kidney stress and end-organ injury (17, 18). These findings are compatible with haemodynamic unloading and structural remodelling, but they do not establish direct myocardial receptor-mediated effects. Reductions in body weight, blood pressure and circulating volume may explain a substantial proportion of the observed changes.

The HFpEF evidence for semaglutide and tirzepatide is complementary but not directly comparative. Semaglutide has replicated symptomatic and functional benefits, whereas tirzepatide has additionally reduced worsening HF events; however, no head-to-head HFpEF trial has compared the two agents. Their respective evidence profiles and limitations are summarised in Table 2.

A claims-based trial-emulation study provided supportive information on the use of semaglutide and tirzepatide in routine-practice HFpEF populations and reported lower rates of heart failure hospitalisation or all-cause death than with sitagliptin (45). However, the analysis did not reproduce random treatment allocation, used an active diabetes comparator rather than placebo and remained vulnerable to residual confounding. It therefore informs transportability but does not establish comparative efficacy between semaglutide and tirzepatide or replace the randomised evidence from STEP-HFpEF and SUMMIT. Observational findings in non-obese HFpEF should likewise be considered hypothesis-generating until confirmed in dedicated randomised trials (46).

### 3.4 Limits of extrapolation to HFrEF and advanced heart failure

The favourable findings in obesity-related HFpEF should not be interpreted as evidence of a uniform heart-failure benefit across ejection-fraction categories or clinical phenotypes. STEP-HFpEF and STEP-HFpEF DM demonstrated improvements in symptoms, physical function and body weight in obesity-related HFpEF, whereas SUMMIT additionally demonstrated fewer worsening heart failure events (10–18). These trials enrolled patients in whom obesity was an important component of the HFpEF phenotype and do not establish efficacy in lean, infiltrative, valvular or other non-obesity-related forms of HFpEF.

Evidence in heart failure with reduced ejection fraction (HFrEF) is less favourable and remains clinically uncertain. In FIGHT, liraglutide did not improve post-hospitalisation clinical stability in patients with advanced HFrEF. In LIVE, liraglutide did not improve left-ventricular systolic function and was associated with an increase in heart rate and a higher frequency of serious cardiac events (47, 48). These studies were smaller than contemporary obesity-related HFpEF trials and evaluated a different agent and patient population, but their findings argue against extrapolating the HFpEF results to HFrEF as a general class effect.

Accordingly, incretin-based therapy in patients with HFrEF should currently be prescribed for a supported obesity, diabetes or atherosclerotic cardiovascular indication rather than as established disease-modifying HFrEF therapy. Guideline-directed HFrEF treatment remains foundational. Particular caution is warranted in advanced heart failure, hypotension, renal dysfunction, cachexia or frailty, where gastrointestinal intolerance and loss of lean mass may reduce the net clinical benefit.

## 4 Mechanisms of cardiovascular protection: evidence hierarchy and remaining uncertainty

Cardiovascular benefit from incretin-based therapy probably arises from a convergent network rather than a single pathway. Randomised human trials demonstrate effects on body weight, glycaemia, blood pressure, albuminuria, inflammatory biomarkers and clinical outcomes, but these findings do not by themselves establish which changes causally mediate cardiovascular benefit. Human imaging and physiological studies provide supportive evidence for changes in cardiac loading, ectopic adiposity, vascular function and myocardial perfusion. By contrast, direct cardiomyocyte receptor signalling, ferroptosis, apoptosis and several endothelial or intracellular pathways remain supported mainly by preclinical or *ex vivo* evidence. The following discussion is therefore organised according to the maturity of the available evidence. The evidence hierarchy for the principal proposed cardiovascular mechanisms is summarised in Table 3. These interconnected mechanisms and their potential cardiovascular consequences are summarized in Figure 1.

**Table 3:** Evidence hierarchy for proposed cardiovascular mechanisms of GLP-1-based therapies.

Mechanistic domain	Principal observations	Main evidence source	Evidence interpretation	Key limitation
Body weight and adiposity reduction	Reduced body weight, visceral adiposity and obesity-related symptom burden	Randomised human intervention trials	Directly demonstrated human treatment effect; likely an important contributor to benefit	Achieved weight loss is a post-randomisation variable and does not establish exclusive causal mediation
Haemodynamic unloading and congestion	Reduced diuretic requirements, circulatory overload and left-ventricular mass in obesity-related HFpEF	Randomised trials, secondary analyses and human imaging	Supportive human evidence for reduced loading conditions	Cannot distinguish effects of weight loss, blood pressure reduction, sodium intake and direct drug actions
Systemic inflammation	Reduced C-reactive protein and inflammatory burden	Randomised human biomarker data	Established biomarker response	C-reactive protein is nonspecific and has not been validated as a causal mediator or treatment-selection biomarker
Kidney–heart interactions	Reduced albuminuria, kidney disease progression and cardiovascular death	Dedicated randomised kidney outcome evidence	Robust clinical outcome evidence for cardiorenal benefit	The relative contributions of systemic and direct renal mechanisms remain uncertain
Endothelial and vascular function	Changes in selected measures of vascular function	Small randomised or physiological human studies	Supportive human mechanistic evidence	Findings are influenced by background therapy and have not been linked directly to cardiovascular outcome mediation
Coronary microvascular function and myocardial energetics	Improved myocardial perfusion, energetics or exercise tolerance in limited human studies	Small human physiological studies and experimental models	Translationally supportive but not clinically definitive	No established indication for coronary microvascular dysfunction or residual angina
Cardiac-adjacent adipose tissue	Reduced paracardiac adipose tissue; receptor expression reported in human epicardial fat	Human imaging and tissue studies	Supports biological relevance of ectopic adiposity	A receptor-specific local cardiac effect has not been established
Plaque biology and stabilisation	Reduced macrophage activation, oxidative stress and lesion progression	Predominantly animal and cellular studies	Biologically plausible preclinical mechanism	Cardiovascular outcome trials do not directly demonstrate changes in human plaque composition
Direct myocardial receptor signalling	Potential functional myocardial responses and cardioprotection	*Ex vivo* human tissue and animal studies	Investigational mechanism	Functionally meaningful GLP-1 receptor expression in adult human ventricular cardiomyocytes remains unresolved
AMPK–eNOS signalling, apoptosis and ferroptosis	Reduced ischaemia–reperfusion injury, apoptosis or ferroptosis in experimental models	Predominantly animal and cellular studies	Preclinical hypothesis	No direct validation as a mediator of cardiovascular outcome benefit in humans

AMPK, AMP-activated protein kinase; eNOS, endothelial nitric oxide synthase; GLP-1, glucagon-like peptide-1; HFpEF, heart failure with preserved ejection fraction.

Evidence categories describe the principal source and maturity of the available evidence and do not represent a formal GRADE assessment. Demonstration of a clinical outcome or intermediate phenotype does not establish causal mediation by an individual pathway.

**Figure 1:**
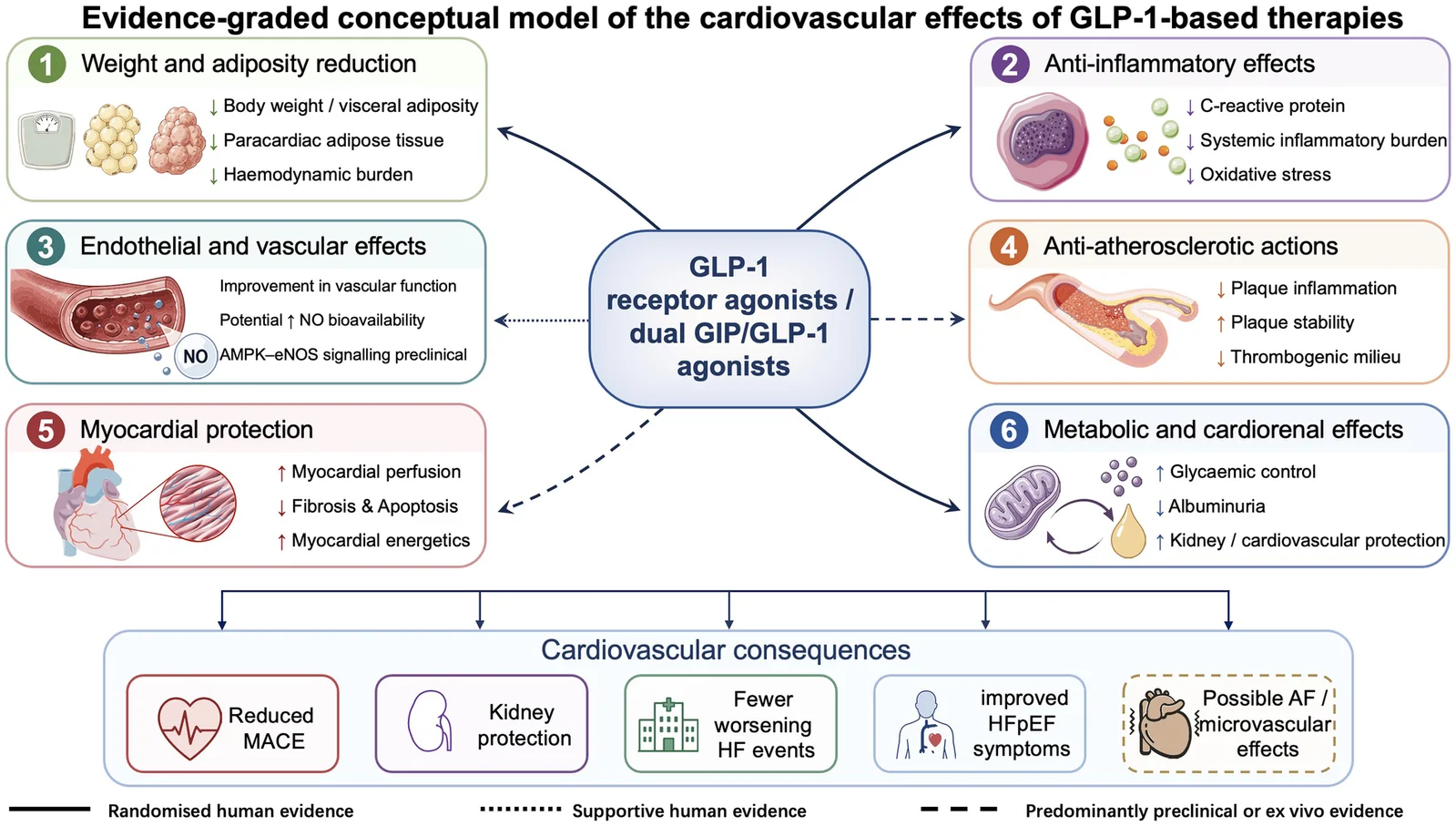
Evidence-graded mechanistic framework for cardiovascular effects of GLP-1-based therapies. This author-generated conceptual model integrates clinical, translational and preclinical evidence concerning the potential cardiovascular effects of GLP-1 receptor agonists and dual GIP/GLP-1 receptor agonism. Randomised human studies directly demonstrate reductions in body weight, glycaemia, blood pressure, albuminuria and inflammatory biomarkers, together with improvements in selected cardiovascular and kidney outcomes. Human imaging and physiological studies provide supportive evidence for changes in cardiac loading, ectopic adiposity, vascular function and myocardial perfusion. Direct cardiomyocyte receptor signalling, plaque-cell biology, apoptosis, ferroptosis and specific intracellular pathways remain supported predominantly by preclinical or *ex vivo* studies. Solid arrows indicate treatment effects demonstrated in randomised human studies; dotted arrows indicate supportive human imaging, biomarker or physiological evidence; and dashed arrows indicate predominantly preclinical or *ex vivo* hypotheses. The diagram illustrates potential biological relationships and does not establish causal mediation by any individual pathway. AMPK, AMP-activated protein kinase; CRP, C-reactive protein; eNOS, endothelial nitric oxide synthase; GIP, glucose-dependent insulinotropic polypeptide; GLP-1, glucagon-like peptide-1; HFpEF, heart failure with preserved ejection fraction; MACE, major adverse cardiovascular events; NO, nitric oxide.

### 4.1 Adiposity reduction and hemodynamic unloading

Adiposity reduction and haemodynamic unloading are among the mechanisms most directly supported by human intervention studies. In STEP-HFpEF, greater achieved weight loss was associated with larger improvements in health status, and semaglutide reduced body weight, inflammatory burden and diuretic requirements (10–15). In SUMMIT, tirzepatide reduced body weight and worsening heart failure events, while cardiac magnetic resonance imaging demonstrated reductions in left-ventricular mass and paracardiac adipose tissue (16–18). These findings support the clinical importance of reducing obesity-related loading conditions, but associations between achieved weight loss and outcome improvement do not prove that weight loss is the sole causal mediator.

Reduction of visceral and ectopic fat may improve insulin sensitivity, inflammatory signalling, blood pressure, plasma-volume distribution and the mechanical burden of exercise. Epicardial and paracardiac fat are of particular interest because of their proximity to the myocardium and coronary arteries. However, although human studies demonstrate that these fat depots can be reduced, the functional importance of incretin-receptor expression within adipose tissue and any receptor-specific local cardiac effect remain uncertain (17, 49).

Body composition is also relevant to the balance between benefit and risk. Pharmacological weight reduction may include loss of lean mass, particularly in older or frail patients. A randomised study found that combining exercise with GLP-1RA therapy improved abdominal adiposity and inflammatory measures more than either intervention alone (50). These data support preservation of muscle through adequate nutrition and resistance exercise, but do not establish skeletal-muscle change as a mediator of cardiovascular outcome benefit.

### 4.2 Inflammation, endothelial function and atherosclerotic risk

Randomised clinical trials consistently demonstrate modest reductions in blood pressure and inflammatory biomarkers with GLP-1-based therapy, and substantial reductions in C-reactive protein were observed in the STEP-HFpEF programme (10–12). These findings support improvement in the systemic metabolic and inflammatory environment. However, C-reactive protein is a nonspecific downstream marker, and it has not been validated as a treatment-selection biomarker or established as a causal mediator of cardiovascular benefit.

Human studies provide limited but supportive evidence for improved vascular function. In a randomised trial, semaglutide and empagliflozin exerted separable and combined effects on vascular measures (51). Nevertheless, endothelial function is influenced by background lipid-lowering, renin–angiotensin system blockade, smoking, blood pressure and glycaemic control, making it difficult to attribute vascular changes to a specific receptor-mediated pathway.

Plaque stabilisation, reduced macrophage activation, increased nitric-oxide bioavailability and attenuation of vascular oxidative stress are supported predominantly by experimental studies. Cardiovascular outcome trials demonstrate fewer clinical events but do not directly establish changes in human plaque composition. Accordingly, anti-inflammatory, endothelial and plaque effects should be described as biologically plausible or supported by translational evidence rather than as fully validated mechanisms of MACE reduction.

### 4.3 Myocardial receptor uncertainty and potential direct cardiac effects

Whether functionally meaningful GLP-1 receptor expression is present in adult human ventricular cardiomyocytes remains unresolved. Experimental studies have reported myocardial protection, improved perfusion and *ex vivo* functional responses, but these findings do not establish that the clinical cardiovascular benefits of GLP-1RAs are mediated through direct receptor activation in cardiomyocytes (52–56). Some effects may instead arise through endothelial or other non-myocyte cell populations, autonomic regulation, kidney function, reduced adiposity or changes in cardiac loading conditions.

Interpretation is further limited by differences between species, experimental models and clinically relevant drug exposure. Evidence from isolated myocardium or animal ischaemia–reperfusion models cannot reproduce the systemic metabolic, renal, neurohumoral and haemodynamic effects present in treated patients. Conversely, cardiovascular outcome trials demonstrate clinical benefit but cannot identify the responsible cardiac cell type or distinguish direct myocardial signalling from indirect systemic mechanisms.

Small human physiological studies provide supportive evidence that incretin-based therapy may influence myocardial perfusion and energetics (57). However, effects involving AMPK–eNOS signalling, cardiomyocyte apoptosis, ferroptosis and direct myocardial receptor activation remain predominantly preclinical or *ex vivo* (52–56). These findings should therefore be presented as mechanistic hypotheses rather than established explanations for clinical event reduction and do not currently support specific indications for myocardial salvage, residual angina or coronary microvascular dysfunction.

### 4.4 Kidney–heart interactions

FLOW demonstrated that semaglutide reduces clinically important kidney outcomes and cardiovascular death in patients with type 2 diabetes and chronic kidney disease (29). Proposed explanations include improvements in glycaemia, blood pressure, body weight, albuminuria and systemic inflammation. However, the trial established clinical efficacy rather than a specific renal cellular mechanism and does not determine the relative contribution of direct renal signalling vs. systemic metabolic and haemodynamic effects.

These benefits complement, rather than replace, renin–angiotensin system blockade, SGLT2 inhibition and finerenone when indicated (25, 30–32, 58–60). Tirzepatide has shown favourable albuminuria and estimated glomerular filtration rate (eGFR) signals in *post hoc* analyses, but dedicated kidney outcome evidence remains less mature (61). Observational and systematic-review findings provide supportive evidence but should not supersede randomised outcome trials (62, 63).

Overall, the mechanistic evidence is strongest for systemic metabolic, adiposity-related, haemodynamic and cardiorenal effects demonstrated in human studies; intermediate for vascular, imaging and physiological changes; and weakest for direct cardiomyocyte and intracellular pathways. Clinical conclusions should follow this hierarchy and should not attribute cardiovascular protection to a single experimentally identified mechanism.

## 5 Atrial fibrillation and stroke

### 5.1 Atrial remodelling and rhythm outcomes

Obesity promotes AF through atrial stretch, epicardial fat inflammation, sleep apnoea, hypertension and autonomic imbalance. Weight reduction can lower AF burden and improve rhythm-control outcomes, so a signal with semaglutide is biologically credible. The meta-analysis reporting a 42% relative reduction in incident AF was based on events collected across trials not designed primarily for rhythm outcomes (64). Event ascertainment, monitoring intensity and AF definitions therefore varied, and the result should be regarded as a strong hypothesis rather than a definitive antiarrhythmic indication.

Dedicated studies with prespecified rhythm endpoints are needed to determine whether incretin-based therapy reduces incident AF, recurrent arrhythmia or overall AF burden. Until such evidence is available, these agents should be regarded as components of comprehensive cardiometabolic risk-factor management rather than as established antiarrhythmic therapies.

### 5.2 Stroke, peripheral arterial disease and microvascular disease

Stroke reduction is one of the more consistent components of GLP-1RA cardiovascular benefit, especially in diabetes populations. The pathway is likely multifactorial: lower blood pressure and atherogenic lipoproteins, improved endothelial function, less inflammation and fewer atherosclerotic events all contribute. Whether AF prevention adds a measurable cardioembolic component is unknown. Trials have not been designed to separate large-artery, small-vessel and cardioembolic stroke mechanisms, and no evidence supports changing antithrombotic treatment because an incretin drug is prescribed.

Peripheral arterial disease may represent a high-absolute-risk phenotype, but dedicated randomised limb-outcome evidence remains limited. In patients with diabetes and previous major adverse limb events, GLP-1RA use was associated with lower rates of recurrent limb events, amputation, MACE, dialysis and death than DPP-4 inhibitor use (65). Despite active-comparator weighting, residual confounding cannot be excluded. These findings are hypothesis-generating and support further investigation; current treatment decisions should continue to rely primarily on established cardiovascular, glycaemic and limb-care indications rather than on this observational comparison alone.

Microvascular effects are less uniform. Evidence is strongest for albuminuria and kidney outcomes, whereas data for neuropathy, cerebral small-vessel disease, coronary microvascular angina and autonomic neuropathy remain limited (66). Rapid improvement in glycaemia can transiently worsen diabetic retinopathy in susceptible patients, particularly those with advanced baseline disease. This phenomenon reflects the speed of glucose reduction rather than a proven toxic retinal effect of the molecule, but it justifies retinal assessment and coordinated diabetes care when HbA1c is markedly elevated.

## 6 Clinical integration in contemporary cardiology

### 6.1 A practical phenotype-directed treatment algorithm

A practical treatment algorithm should begin by identifying the dominant cardiovascular–kidney–metabolic phenotype rather than by selecting the agent associated with the greatest average weight loss. The first step is to determine whether the patient has an evidence-supported clinical setting: established ASCVD with overweight or obesity, type 2 diabetes with ASCVD, type 2 diabetes with chronic kidney disease, or obesity-related HFpEF. Patients with lean HFpEF, advanced HFrEF, low-risk primary-prevention obesity or other unstudied phenotypes should not be assumed to derive the same cardiovascular benefit.

For established ASCVD with overweight or obesity but without diabetes, semaglutide 2.4 mg has direct randomised secondary-prevention evidence from SELECT (7). In type 2 diabetes with established ASCVD, several long-acting GLP-1RAs reduce MACE, oral semaglutide is supported by SOUL, and tirzepatide is noninferior to dulaglutide in SURPASS-CVOT (1–6, 19–23). In obesity-related HFpEF, semaglutide improves symptoms and physical function, whereas tirzepatide additionally reduces worsening heart failure events (10–18). In type 2 diabetes with chronic kidney disease, semaglutide has dedicated kidney outcome evidence from FLOW (29).

The second step is to determine whether an SGLT2 inhibitor is already indicated or prescribed. SGLT2 inhibitors remain foundational when heart failure or chronic kidney disease is a major component of the phenotype. GLP-1-based therapy may then be added when obesity, residual atherosclerotic risk or inadequate metabolic control remains clinically important. In patients with ASCVD and obesity but without heart failure or chronic kidney disease, semaglutide may reasonably be prioritised. When multiple indications coexist, use of both classes should be based on the independent evidence-supported indication for each therapy rather than an assumed synergistic effect.

The third step is to assess patient-specific suitability. Relevant considerations include gastrointestinal tolerability, volume status, kidney function, diabetic retinopathy risk, frailty, sarcopenia, nutritional reserve, route of administration, treatment access, affordability and the likelihood of long-term persistence. These factors may influence whether treatment should be initiated, the rate of dose escalation and the most appropriate agent or formulation.

Finally, treatment response should be assessed according to the original therapeutic objective. Monitoring may include recurrent cardiovascular events, HF symptoms, KCCQ score, physical function, congestion, kidney trajectory, albuminuria, blood pressure, glycaemic control, body weight, nutritional status and adverse effects. Lack of trial-average weight loss does not necessarily indicate treatment failure when cardiovascular, kidney or functional outcomes improve. Conversely, substantial weight loss should not be considered a substitute for guideline-directed lipid-lowering, antithrombotic, SGLT2 inhibitor or heart-failure therapy. The evidence map and proposed clinical framework are summarised in Figures 2, 3. The evidence-supported applications, anticipated benefits and principal limitations of GLP-1-based therapies across major cardiovascular and cardiorenal phenotypes are summarised in Table 4.

**Figure 2:**
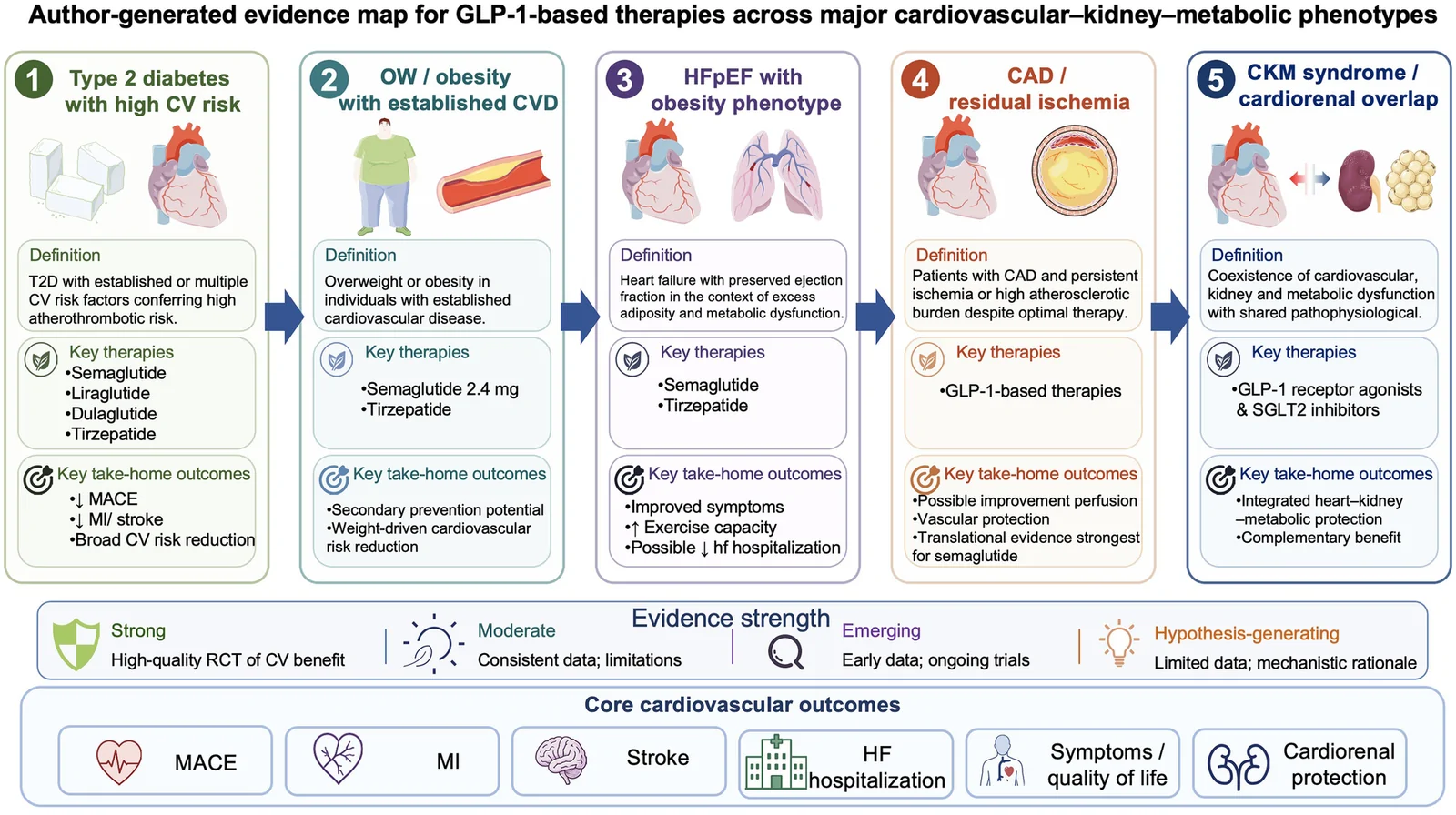
Author-generated evidence map for GLP-1-based therapies across major cardiovascular–kidney–metabolic phenotypes. This author-generated evidence map summarises the strength and nature of the available clinical evidence across major cardiovascular–kidney–metabolic phenotypes. In high-risk type 2 diabetes, several long-acting GLP-1 receptor agonists reduce three-point MACE. In adults with established ASCVD and overweight or obesity without diabetes, semaglutide reduces major cardiovascular events. In obesity-related HFpEF, semaglutide improves symptoms and physical function, whereas tirzepatide additionally reduces worsening heart failure events. Dedicated randomised evidence remains limited or absent for HFrEF, atrial-fibrillation-specific treatment, coronary microvascular dysfunction and low-risk primary prevention. Categories identified as established are based primarily on direct randomised outcome evidence. Supportive or emerging categories are based on secondary analyses, observational studies, imaging studies or mechanistic data and should not be interpreted as established treatment indications. This figure is an author-generated summary and does not represent a formal GRADE assessment. ASCVD, atherosclerotic cardiovascular disease; CKD, chronic kidney disease; HFpEF, heart failure with preserved ejection fraction; HFrEF, heart failure with reduced ejection fraction; MACE, major adverse cardiovascular events; T2D, type 2 diabetes.

**Figure 3:**
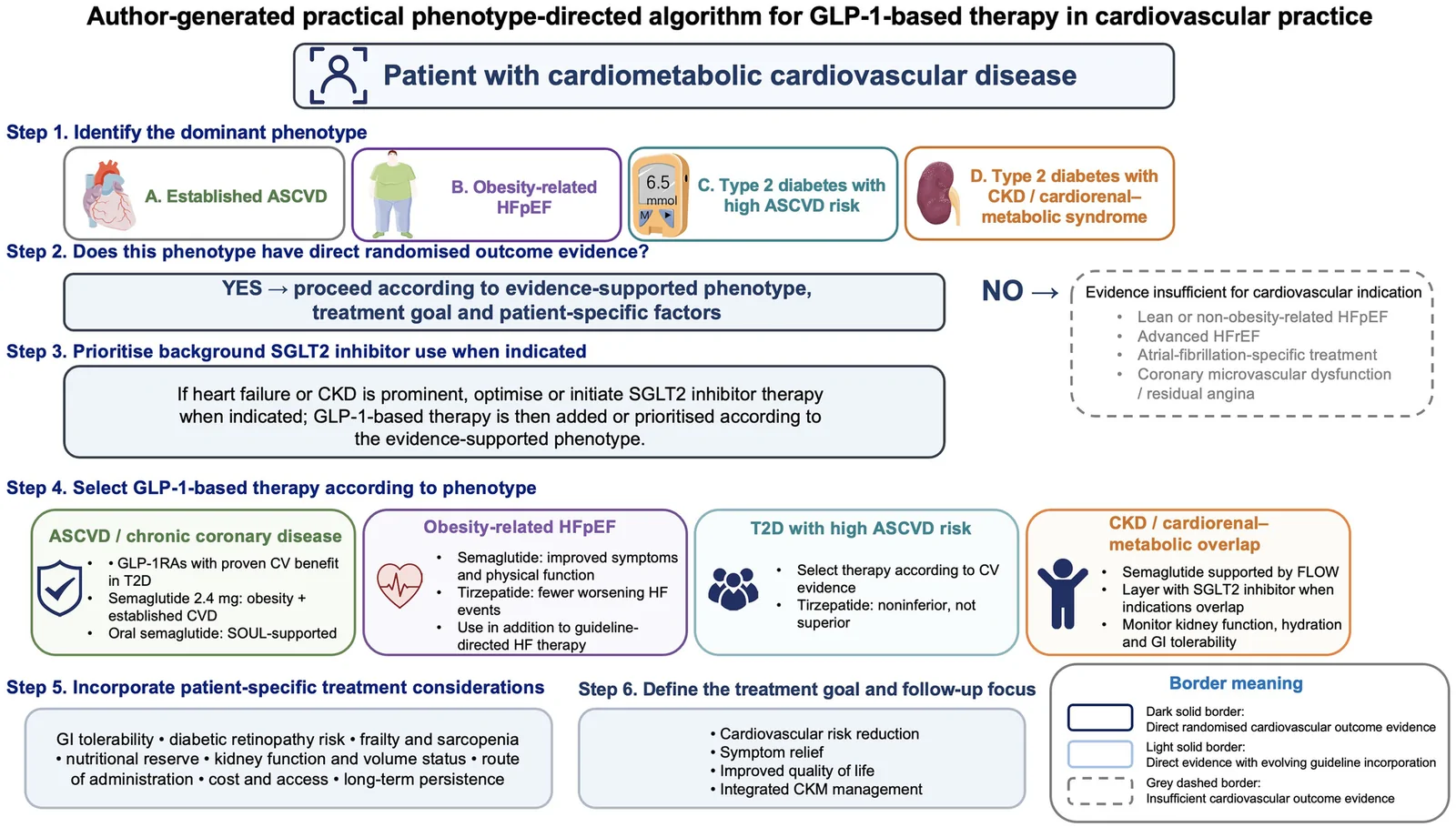
Author-generated practical phenotype-directed algorithm for GLP-1-based therapy in cardiovascular practice. This author-generated algorithm integrates current clinical evidence and patient-specific considerations to illustrate phenotype-directed use of GLP-1-based therapies across major cardiovascular–kidney–metabolic phenotypes. When heart failure or chronic kidney disease is prominent, SGLT2 inhibitor therapy remains foundational when indicated, while GLP-1-based therapy is considered according to phenotype-specific evidence, treatment goals, and individual characteristics, including tolerability, frailty, sarcopenia, nutritional status, kidney function, cost, and treatment persistence. Evidence categories are author-generated and do not represent a formal grading system. Dark solid borders indicate direct randomised cardiovascular outcome evidence, light solid borders indicate emerging clinical evidence with evolving guideline incorporation, dashed borders indicate author-integrated or hypothesis-generating frameworks, and grey dashed borders indicate insufficient cardiovascular outcome evidence. This algorithm is conceptual and should not replace clinical guidelines or individualised treatment decisions. ASCVD, atherosclerotic cardiovascular disease; CKD, chronic kidney disease; CV, cardiovascular; GLP-1, glucagon-like peptide-1; HFpEF, heart failure with preserved ejection fraction; HFrEF, heart failure with reduced ejection fraction; MACE, major adverse cardiovascular events; SGLT2, sodium–glucose cotransporter 2; T2D, type 2 diabetes.

**Table 4:** Phenotype-directed application of GLP-1-based therapies in cardiovascular practice.

Phenotype	Goal	Evidence level and basis	Therapy	Benefit	Limitation
T2D + ASCVD	↓ recurrent CV events	Direct randomised cardiovascular outcome evidence	GLP-1RA/oral sema/tirzepatide	↓ MACE, weight, glucose	Not all agents equal
ASCVD + obesity (no T2D)	Secondary prevention	Direct randomised outcome evidence from SELECT	Semaglutide 2.4 mg	↓ MACE, weight	Not for primary prevention
Obesity HFpEF	Improve symptoms	Direct randomised evidence for symptoms and function; randomised HF-event evidence for tirzepatide	Sema or tirzepatide	↑ function, ↓ weight	No head-to-head
T2D + CKD	↓ renal/CV risk	Direct randomised kidney outcome evidence from FLOW	Semaglutide ± SGLT2 inhibitor	↓ CKD progression	Not replacement therapy
Stable HFrEF	Treat comorbidity	No established randomised HFrEF efficacy; neutral or concerning liraglutide trials	Use only if indicated	Metabolic benefit	Caution in advanced HF
AF + obesity/T2D	Risk factor control	Secondary and pooled trial signals; no dedicated rhythm-outcome trial	GLP-1-based therapy	↓ weight	No AF outcome proof
Angina/CMD	Improve perfusion	Small mechanistic and experimental studies only	Research only	Possible benefit	No RCT evidence
Overlap (ASCVD/CKD/HF)	Multiorgan protection	Randomised subgroup evidence supports preserved benefit; incremental combination benefit remains unproven	GLP-1 + SGLT2 inhibitor	Broad benefit	Synergy unclear

AF, atrial fibrillation; ASCVD, atherosclerotic cardiovascular disease; CKD, chronic kidney disease; CVD, cardiovascular disease; GLP-1RA, glucagon-like peptide-1 receptor agonist; HFpEF, heart failure with preserved ejection fraction; HFrEF, heart failure with reduced ejection fraction; KCCQ, Kansas City Cardiomyopathy Questionnaire; MACE, major adverse cardiovascular events; SGLT2, sodium–glucose cotransporter 2; T2D, type 2 diabetes.

Only rows supported by direct randomised outcome evidence should be interpreted as evidence-based clinical applications. Observational, secondary and mechanistic findings are presented to identify potential applications and evidence gaps, not to establish treatment indications.

### 6.2 Sequencing and combination with SGLT2-inhibitors

Randomised subgroup and meta-analytic data indicate that the cardiovascular and kidney benefits of each drug class are generally preserved when the other is used as background therapy (20, 58–60, 67, 68). These findings support compatibility and complementarity, but they do not quantify the incremental benefit of initiating both classes together.

Observational studies have reported lower cardiovascular and kidney event rates with combination treatment than with either class alone (59, 69, 70). However, patients receiving both therapies may differ systematically in access to care, treatment adherence, clinical surveillance and baseline risk. These analyses therefore cannot establish additive or synergistic efficacy. In the absence of a dedicated randomised outcomes comparison, combination treatment should be justified by coexisting evidence-based indications for each class rather than by an assumed supra-additive effect. The complementary effects of GLP-1-based therapies and SGLT2-inhibitors within the cardiovascular-kidney-metabolic axis are illustrated in Figure 4.

**Figure 4:**
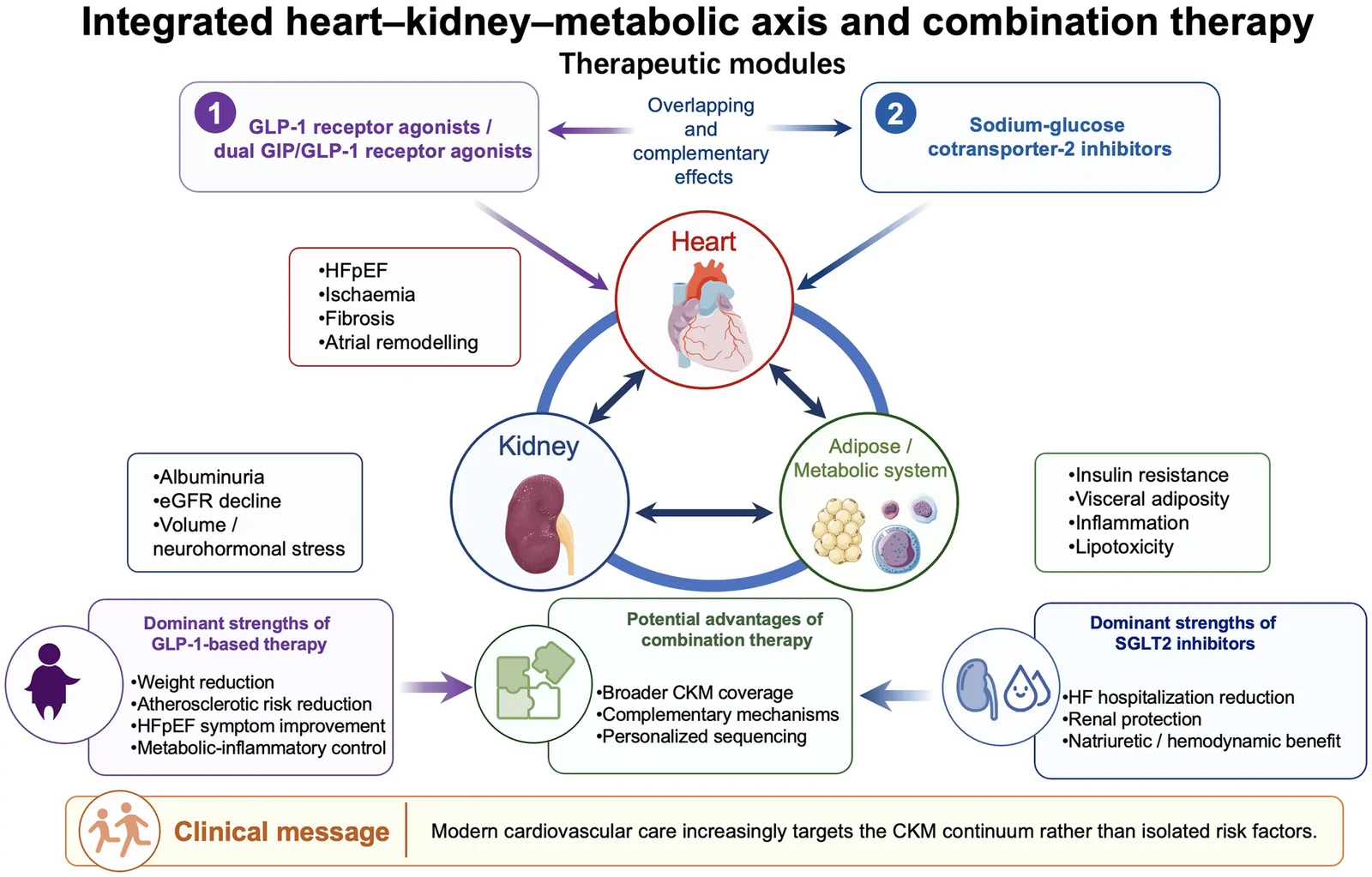
Integrated heart–kidney–metabolic axis and the rationale for combination therapy. This author-generated conceptual synthesis illustrates the partially distinct and potentially complementary roles of GLP-1-based therapies and SGLT2 inhibitors. Cardiovascular disease, kidney dysfunction and metabolic abnormalities interact bidirectionally through shared mechanisms including insulin resistance, visceral adiposity, systemic inflammation, lipotoxicity, haemodynamic stress and neurohormonal activation. Within this interconnected axis, GLP-1-based therapies preferentially address weight excess, residual atherosclerotic risk, inflammatory-metabolic dysfunction and symptoms associated with obesity-related heart failure with preserved ejection fraction. By contrast, sodium-glucose cotransporter-2 inhibitors provide particularly strong benefits for heart failure hospitalization, kidney protection and haemodynamic regulation. Their overlapping but complementary actions provide a biological and clinical rationale for combination treatment in selected patients with cardiovascular–kidney–metabolic syndrome. CKM, cardiovascular–kidney–metabolic; eGFR, estimated glomerular filtration rate; GIP, glucose-dependent insulinotropic polypeptide; GLP-1, glucagon-like peptide-1; HF, heart failure; HFpEF, heart failure with preserved ejection fraction; SGLT2, sodium-glucose cotransporter-2.

### 6.3 Current international guideline recommendations and evidence-to-guideline transition

Major international societies increasingly position GLP-1 receptor agonists according to cardiovascular, kidney and metabolic risk rather than glucose-lowering requirements alone. Across current guidance, the clearest consensus applies to patients with type 2 diabetes and established atherosclerotic cardiovascular disease: a GLP-1RA with demonstrated cardiovascular benefit should be incorporated into comprehensive risk-reduction therapy, whereas SGLT2 inhibitors remain particularly important when heart failure or chronic kidney disease is the dominant phenotype. However, guideline recommendations are not fully synchronized because publication cycles differ and several pivotal trials were reported after the evidence-review periods of earlier documents.

The 2023 ESC Guidelines recommend GLP-1RAs with proven cardiovascular benefit and/or SGLT2 inhibitors in patients with type 2 diabetes and established ASCVD, independent of baseline or target HbA1c and in addition to standard antithrombotic, lipid-lowering and blood-pressure treatment (71). For patients with diabetes and heart failure, SGLT2 inhibitors remain the principal outcome-modifying glucose-lowering class across ejection-fraction categories. The 2024 ESC Guidelines for chronic coronary syndromes further state that semaglutide should be considered in patients with chronic coronary syndrome and overweight or obesity without diabetes to reduce cardiovascular death, myocardial infarction or stroke (72). This recommendation reflects the incorporation of SELECT into European secondary-prevention guidance. The ESC obesity consensus statement similarly identifies semaglutide 2.4 mg as the incretin-based regimen with direct cardiovascular outcome evidence in established cardiovascular disease without type 2 diabetes (73).

The 2023 AHA/ACC multisociety guideline for chronic coronary disease recommends an SGLT2 inhibitor or GLP-1RA with proven cardiovascular benefit in patients with chronic coronary disease and type 2 diabetes to reduce MACE. It also supports GLP-1RA therapy as part of pharmacological weight management in selected patients with chronic coronary disease and overweight or obesity (74). More recent heart-failure evidence has evolved beyond that guideline cycle. The 2026 ACC Expert Consensus Decision Pathway for HFpEF now incorporates incretin-based therapies into phenotype-directed management and recognizes visceral adiposity, inflammation and metabolic dysfunction as important therapeutic substrates. This document is an expert consensus pathway rather than a formal joint AHA/ACC clinical practice guideline, and that distinction should be maintained when interpreting its recommendations (75).

The ADA Standards of Care in Diabetes—2026 recommend GLP-1RAs with demonstrated cardiovascular benefit in people with type 2 diabetes and established ASCVD, multiple ASCVD risk factors or chronic kidney disease. SGLT2 inhibitors and GLP-1RAs are positioned as core cardiovascular–kidney risk-reduction therapies rather than solely as agents for achieving glycaemic targets, and combination therapy may be considered when evidence-supported indications for both classes coexist (76). KDIGO guidance remains more sequential: in adults with type 2 diabetes and CKD who have not achieved individualised glycaemic targets despite metformin and SGLT2 inhibitor treatment, or who cannot use those therapies, a long-acting GLP-1RA is recommended, with preference for agents with documented cardiovascular benefit (77).

Recent trials are likely to continue reshaping these recommendations. SELECT has already influenced guidance for secondary prevention in obesity without diabetes, whereas FLOW has strengthened the kidney rationale for semaglutide beyond earlier albuminuria-based evidence. KDIGO commentary published after FLOW concluded that the trial reinforces the existing recommendation and raises the question of whether GLP-1RA use in type 2 diabetes and CKD should extend beyond patients requiring additional glycaemic control, although broader use has not yet become a formal KDIGO recommendation. SOUL and SURPASS-CVOT refine agent-specific ASCVD evidence, while STEP-HFpEF and SUMMIT have moved obesity-related HFpEF from a mechanistically plausible indication toward an evidence-supported clinical phenotype (75, 78). Current international recommendations and major evidence-to-guideline gaps are summarised in Table 5.

**Table 5:** International guideline and consensus recommendations for GLP-1-based therapies in cardiovascular and kidney disease.

Society and document	Population or phenotype	Current position of GLP-1-based therapy	Position of SGLT2 inhibitors	Evidence-to-guideline gap
2023 ESC Guidelines for CVD in diabetes	T2D with established ASCVD	GLP-1RA with proven CV benefit is recommended to reduce CV events, independent of glycaemic control and in addition to standard care	Recommended particularly when HF or CKD is present	Published before SOUL and SURPASS-CVOT
2024 ESC Guidelines for chronic coronary syndromes	Chronic coronary syndrome with overweight or obesity without diabetes	Semaglutide should be considered to reduce CV death, MI or stroke	Used when a separate HF, CKD or diabetes indication exists	Direct recommendation is currently specific to semaglutide evidence from SELECT
2024 ESC obesity consensus statement	Obesity with cardiovascular disease	Supports evidence-based obesity treatment; semaglutide 2.4 mg has direct CV outcome evidence in established CVD without T2D	Consider according to accompanying HF, CKD or diabetes	Consensus statement rather than a separate obesity clinical practice guideline
2023 AHA/ACC multisociety chronic coronary disease guideline	Chronic coronary disease with T2D	GLP-1RA with proven CV benefit is recommended to reduce MACE	SGLT2 inhibitor is also recommended; prioritised when HF is prominent	Predates SOUL, SURPASS-CVOT, FLOW and SUMMIT
2026 ACC HFpEF Expert Consensus Decision Pathway	HFpEF, particularly obesity-related and metabolically driven phenotypes	Incorporates incretin-based therapy into contemporary phenotype-directed HFpEF management	Remains a principal foundational therapy for HFpEF	Expert consensus pathway, not a formal joint AHA/ACC guideline
ADA Standards of Care in Diabetes—2026	T2D with established ASCVD, multiple ASCVD risk factors or CKD	GLP-1RA with demonstrated CV benefit is recommended as part of comprehensive risk reduction	Recommended for ASCVD, HF or CKD according to phenotype; combination may be considered when indications overlap	Requires agent-specific interpretation; not every GLP-1RA has proven CV benefit
KDIGO 2022 Diabetes in CKD and 2024 CKD Guidelines	T2D with CKD and glycaemic targets unmet despite metformin and SGLT2 inhibition, or inability to use these agents	Recommend a long-acting GLP-1RA; prioritize agents with documented CV benefit	Foundational kidney-protective therapy when tolerated	FLOW strengthens kidney outcome evidence and may support broader future positioning beyond glycaemic need
2025 KDIGO incretin commentary	Diabetes and CKD after FLOW and SELECT	Confirms that current GLP-1RA recommendation remains appropriate and is strengthened by newer RCTs	Continues to support layered treatment	Broader GLP-1RA use is discussed but is not yet a formal guideline recommendation

### 6.4 Safety, tolerability and treatment discontinuation

The clinical value of GLP-1-based therapy depends not only on efficacy but also on whether patients can tolerate dose escalation and sustain treatment. Gastrointestinal adverse effects are the most frequent practical limitation and an important cause of dose reduction, treatment interruption or discontinuation. Nausea, vomiting, diarrhoea and constipation occur most commonly during initiation and escalation and may be mitigated by gradual titration, smaller meals and avoidance of high-fat meals. The highest tolerated dose may be more appropriate than continued escalation when clinically meaningful cardiovascular, metabolic or functional benefit has already been achieved.

Persistent vomiting, diarrhoea or markedly reduced oral intake may lead to dehydration, hypotension, electrolyte abnormalities and acute kidney injury, particularly in patients receiving diuretics, renin–angiotensin system inhibitors or SGLT2 inhibitors. Risk is amplified in older adults, chronic kidney disease and heart failure. Volume status, blood pressure, kidney function and concomitant diuretic treatment should therefore be reassessed during dose escalation and after substantial weight loss. Although GLP-1RAs are not generally nephrotoxic, unexplained deterioration in kidney function or new proteinuria warrants evaluation rather than automatic attribution to volume depletion or diabetic kidney disease (79).

Gallbladder and biliary events occur more frequently during rapid or substantial weight loss. Pancreatitis remains uncommon, and randomised trial evidence has not established a major causal class effect; nevertheless, persistent severe abdominal pain requires prompt assessment and temporary treatment interruption while pancreatitis or biliary disease is excluded. A previous history of pancreatitis should prompt individualised risk assessment rather than automatic exclusion in all patients. Contraindications relating to medullary thyroid carcinoma or multiple endocrine neoplasia type 2 should be observed according to the prescribing information for the specific agent.

Diabetic retinopathy requires particular attention when a large and rapid reduction in HbA1c is anticipated. Early worsening is most relevant in patients with long-standing poor glycaemic control and pre-existing proliferative or severe non-proliferative retinopathy and appears to be associated primarily with the speed of glycaemic improvement rather than established direct retinal toxicity (66). Baseline ophthalmic assessment, coordinated retinal follow-up and appropriate adjustment of insulin or sulfonylurea therapy should be considered in high-risk patients.

Nutritional status and body composition are especially important in older adults, frail patients and those with advanced heart failure, cancer or recent unintentional weight loss. Pharmacological weight reduction includes some loss of lean mass, and gastrointestinal intolerance or excessive caloric restriction may worsen sarcopenia, weakness and functional decline. Assessment should therefore extend beyond body weight to appetite, dietary protein intake, muscle strength, gait or physical performance and recent weight trajectory. Resistance exercise and adequate protein intake should accompany treatment whenever feasible. In advanced HFrEF, cachexia, symptomatic hypotension or limited cardiac reserve, treatment should be initiated cautiously and continued only when the metabolic or cardiovascular benefit clearly exceeds the nutritional and haemodynamic risk (47, 48).

Periprocedural management should be individualised because delayed gastric emptying may increase aspiration risk during anaesthesia or deep sedation, whereas unnecessary prolonged interruption may destabilize glycaemia and chronic weight management. Decisions should consider the specific agent, dosing interval, current gastrointestinal symptoms, procedure type and anaesthetic plan in accordance with current local guidance. After acute myocardial infarction, heart failure hospitalisation or major surgery, initiation or reinitiation is generally most appropriate once haemodynamics, kidney function and oral intake have stabilised.

The principal safety, tolerability and implementation considerations relevant to cardiovascular practice are summarised in Table 6.

**Table 6:** Safety, tolerability and implementation considerations for GLP-1-based therapy in cardiovascular practice.

Clinical issue	Principal concern	Higher-risk settings	Practical considerations
Gastrointestinal intolerance	Nausea, vomiting, diarrhoea, constipation, reduced oral intake and treatment discontinuation	Rapid dose escalation, older age, frailty, CKD, HF and concomitant polypharmacy	Gradual titration; smaller meals; avoid high-fat meals; consider maintaining the highest tolerated dose rather than mandatory escalation
Volume depletion and kidney dysfunction	Hypotension, electrolyte abnormalities and acute kidney injury during vomiting or poor intake	Diuretic, SGLT2 inhibitor or renin–angiotensin system inhibitor use; CKD; HF; older adults	Reassess volume status, blood pressure, kidney function and diuretic dose; provide sick-day guidance
Gallbladder and biliary disease	Increased biliary events during rapid or substantial weight loss	Previous gallstones, rapid weight reduction and recurrent abdominal symptoms	Assess persistent right-upper-quadrant or severe abdominal pain; investigate clinically suspected biliary disease
Pancreatitis	Uncommon but clinically important differential diagnosis for persistent severe abdominal pain	Previous pancreatitis or biliary disease	Temporarily interrupt treatment during evaluation; individualize decisions in patients with previous pancreatitis
Diabetic retinopathy	Early worsening during rapid glycaemic improvement	Advanced baseline retinopathy, long-standing poor glycaemic control and large anticipated HbA1c reduction	Consider baseline retinal assessment and coordinated follow-up; adjust insulin or sulfonylurea therapy when appropriate
Frailty, sarcopenia and nutritional risk	Loss of lean mass, weakness, functional decline and malnutrition	Older adults, advanced HF, cancer, low protein intake and recent unintentional weight loss	Assess appetite, weight trajectory, muscle strength and physical function; support adequate protein intake and resistance exercise
Advanced HFrEF	Gastrointestinal intolerance, tachycardia, hypotension, cachexia and reduced physiological reserve	Advanced or recently decompensated HFrEF	Use only for a supported metabolic or ASCVD indication; initiate cautiously and monitor nutrition, heart rate, volume status and symptoms
Periprocedural treatment	Delayed gastric emptying and possible aspiration risk	Active gastrointestinal symptoms, anaesthesia or deep sedation	Individualize interruption according to the agent, dose timing, symptoms, procedure and local guidance
Treatment discontinuation	Appetite recurrence, weight regain and deterioration of metabolic risk	Adverse effects, medication shortages, insurance changes and poor follow-up	Discuss long-term treatment before initiation; establish a transition or alternative-treatment plan when discontinuation is unavoidable
Economic and access barriers	Delayed initiation, incomplete dose escalation, interrupted therapy and inequitable treatment	High medication cost, limited insurance coverage and supply constraints	Prioritize evidence-supported high-risk phenotypes; assess financial sustainability and available alternatives before initiation

ASCVD, atherosclerotic cardiovascular disease; CKD, chronic kidney disease; GLP-1, glucagon-like peptide-1; HbA1c, glycated haemoglobin; HF, heart failure; HFrEF, heart failure with reduced ejection fraction; SGLT2, sodium–glucose cotransporter 2. Risk assessment and management should be individualised according to the specific agent, local prescribing information, concomitant therapy and current clinical guidance.

### 6.5 Access, persistence and real-world implementation

TAffordability and access are major determinants of real-world cardiovascular effectiveness. Patients with the greatest burden of obesity, diabetes, chronic kidney disease and cardiovascular disease may also face the greatest financial and structural barriers to long-term treatment. Risk-based prioritization may improve allocation of limited resources, but should be based on evidence-supported indications and absolute clinical risk rather than socioeconomic advantage or access to advanced imaging.

Treatment persistence is essential because obesity and cardiometabolic disease are chronic conditions. Interruption or discontinuation is commonly followed by appetite recurrence, weight regain and deterioration of metabolic risk factors. Initiation should therefore include a realistic discussion of long-term treatment, expected adverse effects, financial sustainability and the consequences of discontinuation. Supply interruptions or loss of insurance coverage should trigger consideration of alternative evidence-based strategies rather than unplanned cessation without follow-up.

Cardiology services require explicit pathways for initiation, dose escalation and adverse-effect management. Responsibility should be defined for nutritional assessment, adjustment of insulin and sulfonylureas, monitoring of kidney function and volume status, management of gastrointestinal intolerance and preservation of lean mass. Multidisciplinary collaboration among cardiology, primary care, endocrinology, nephrology, obesity medicine, pharmacy and dietetic services may be required for patients with overlapping cardiovascular–kidney–metabolic disease.

Treatment response should be assessed against the original clinical indication rather than body weight alone. Failure to achieve trial-average weight loss does not necessarily represent treatment failure if cardiovascular risk factors, albuminuria, symptoms, mobility or health status improve. Conversely, substantial weight loss should not be regarded as adequate cardiovascular risk control when lipid-lowering, antithrombotic, antihypertensive or heart-failure therapies remain suboptimal. Periodic review should document achieved benefit, dose tolerance, nutritional and functional status, treatment burden, affordability and willingness to continue.

## 7 Unresolved controversies, conflicting evidence and limits of inference

Despite the favourable results of several contemporary trials, important uncertainties remain. Cardiovascular outcome effects have not been uniform across all GLP-1 receptor agonists; direct myocardial receptor-mediated actions remain unproven; heart-failure findings differ substantially between obesity-related HFpEF and advanced HFrEF; and semaglutide and tirzepatide have not been compared directly in a cardiovascular outcome or HFpEF trial. These limitations should constrain class-wide conclusions and comparative claims.

### 7.1 Is cardiovascular benefit proportional to weight loss?

Weight loss is clearly an important mediator, particularly in obesity-related HFpEF, but it is not a complete surrogate for cardiovascular outcome. SELECT demonstrated event reduction without diabetes, STEP-HFpEF linked greater weight loss to larger symptomatic improvement and SURPASS-CVOT showed substantially greater weight loss with tirzepatide without statistically superior three-point MACE compared with dulaglutide (7, 13, 21). The relationship between weight loss and cardiovascular benefit is therefore neither linear nor uniform across clinical phenotypes.

Mediation analyses can estimate how much benefit is statistically associated with change in weight, HbA1c, blood pressure or albuminuria, but they cannot fully disentangle correlated biological pathways. Weight loss modifies sleep apnoea, inflammation, mobility, plasma volume and adipose signalling simultaneously. Treating it as a single mediator understates its systems-level effects. Conversely, assuming that every kilogram lost confers the same cardiovascular protection ignores drug-specific, population-specific and time-dependent differences.

### 7.2 Can semaglutide and tirzepatide be ranked using indirect evidence?

Tirzepatide produces greater average reductions in body weight and HbA1c than many selective GLP-1RAs, but superior metabolic efficacy is not equivalent to superior cardiovascular efficacy. SURPASS-CVOT demonstrated noninferiority to dulaglutide for three-point MACE and did not demonstrate superiority (21). It also did not compare tirzepatide directly with semaglutide.

Cross-trial comparisons with SELECT, SOUL, STEP-HFpEF or SUMMIT are methodologically unreliable because the populations, comparators, treatment doses, follow-up periods and primary endpoints differed. No direct randomised evidence currently establishes that tirzepatide is superior to semaglutide, or vice versa, for atherosclerotic cardiovascular events or obesity-related HFpEF. Treatment selection should therefore be based on the evidence supporting the specific clinical indication rather than on comparison of numerical results across separate trials.

### 7.3 Are the cardiovascular effects directly myocardial?

Direct myocardial and vascular actions remain biologically plausible but clinically unproven. Experimental studies support effects on perfusion, endothelial signalling, fibrosis and ischaemic injury (52–56), but human outcome trials cannot separate these mechanisms from the consequences of weight loss, lower blood pressure, reduced inflammation, improved kidney function and altered loading conditions. Current clinical recommendations should therefore remain phenotype- and outcome-based rather than mechanism-based.

### 7.4 Can current evidence be generalized?

Generalizability is particularly limited in heart failure. The consistent benefits observed in STEP-HFpEF and SUMMIT apply primarily to obesity-related HFpEF, whereas FIGHT and LIVE did not establish benefit in HFrEF and raised potential tolerability or safety concerns (10–18, 47, 48). Evidence remains insufficient for lean HFpEF, infiltrative cardiomyopathy, advanced HFrEF and patients with cachexia or severe frailty.

The evidence is strongest in patients with overweight or obesity, established cardiovascular disease, type 2 diabetes, chronic kidney disease or obesity-related HFpEF. Data are limited for lean HFpEF, advanced HFrEF, type 1 diabetes, severe frailty and primary prevention in lower-risk obesity. Broader prescribing without phenotype-specific evidence risks diluting benefit and exposing patients to cost and adverse effects without a clear cardiovascular indication.

Generalizability is also limited by trial participation. Older adults with severe frailty, advanced CKD, recent acute coronary syndromes, active cancer or complex multimorbidity are often underrepresented. Real-world studies can assess whether randomised findings are transportable to populations underrepresented in clinical trials, including older adults and patients with multimorbidity. However, they cannot fully resolve treatment-selection bias, differences in dose attainment, discontinuation or competing mortality. They should therefore be used to identify evidence gaps and assess implementation rather than to extend efficacy claims to untested phenotypes.

Primary prevention remains the largest unresolved population question. Obesity is a major lifelong cardiovascular risk factor, but most people with obesity will not experience an event within a short trial horizon. A primary-prevention programme would require long follow-up, careful enrichment and assessment of lifetime benefit, cost and safety. Coronary calcium, kidney disease, diabetes, inflammatory burden and strong family history may help identify higher-risk groups, but no current biomarker is validated to select an incretin therapy specifically.

## 8 Key knowledge gaps and research priorities

Several questions remain despite the expanding evidence base. First, agent-specific and phenotype-specific effects require clarification. Direct randomised comparisons between semaglutide and tirzepatide are lacking, and neutral cardiovascular outcome trials with some GLP-1 receptor agonists caution against assuming uniform efficacy across agents. Comparative research should focus on clinically relevant outcomes rather than weight loss alone.

Second, the relative contributions of weight loss, haemodynamic unloading, kidney protection, inflammation, vascular effects and direct myocardial signalling remain uncertain. Human translational studies are needed to determine which pathways mediate clinical outcomes and whether validated biomarkers can identify differential treatment response. Biomarker and multi-omics findings require external validation before clinical use.

Third, heart-failure evidence remains phenotype-dependent. Benefits are established primarily in obesity-related HFpEF, whereas efficacy and safety in HFrEF and non-obesity-related HFpEF remain uncertain, particularly in frail or cachectic patients (47, 48). Future research should clarify these understudied populations and determine how lean mass and nutritional status influence net benefit.

Fourth, the optimal integration of GLP-1-based therapies with SGLT2 inhibitors and finerenone is not established. Existing subgroup and observational data support complementarity but not proven synergy (58, 60, 68). Further evidence is needed regarding sequencing, incremental benefit and safety in patients with overlapping ASCVD, heart failure, chronic kidney disease and obesity.

Finally, implementation remains a major evidence gap. Long-term persistence, treatment discontinuation, weight regain, dose attainment, affordability, access and multidisciplinary delivery will determine population effectiveness (80–83). Future work should therefore evaluate not only efficacy, but also durability, tolerability and equity.

## 9 Conclusions

Robust evidence now supports phenotype-specific cardiovascular use of GLP-1-based therapies. Several long-acting GLP-1 receptor agonists reduce three-point MACE in high-risk type 2 diabetes. Semaglutide reduces major cardiovascular events in adults with established cardiovascular disease and overweight or obesity without diabetes, oral semaglutide reduces MACE in high-risk type 2 diabetes, and semaglutide improves symptoms and physical function in obesity-related HFpEF and reduces clinically important kidney outcomes in type 2 diabetes with chronic kidney disease. Tirzepatide reduces worsening heart failure events in obesity-related HFpEF and is noninferior to dulaglutide for MACE in patients with type 2 diabetes and established ASCVD. These therapies should be selected according to the dominant cardiovascular–kidney–metabolic phenotype and used in addition to, rather than instead of, established lipid-lowering, antithrombotic, SGLT2 inhibitor, kidney-protective and guideline-directed heart-failure therapies.

Important uncertainties remain. No direct randomised trial has established the overall cardiovascular superiority of tirzepatide over semaglutide, or vice versa. Evidence is insufficient to support cardiovascular indications in lean or non-obesity-related HFpEF, advanced HFrEF, low-risk primary prevention, atrial-fibrillation-specific treatment or coronary microvascular dysfunction. Direct cardiomyocyte receptor signalling, ferroptosis and several endothelial or intracellular pathways remain predominantly translational or preclinical hypotheses rather than established mechanisms of clinical benefit. Current practice should therefore be guided by phenotype-specific randomised outcome evidence, patient tolerance and long-term feasibility—not by weight-loss magnitude, observational comparisons or mechanistic plausibility alone.

## Glossary

6MWD: six-minute walk distance
AF: atrial fibrillation
AMPK: AMP-activated protein kinase
ASCVD: atherosclerotic cardiovascular disease
BMI: body mass index
CAD: coronary artery disease
CKD: chronic kidney disease
CKM: cardiovascular–kidney–metabolic
CMR: cardiac magnetic resonance
CRP: C-reactive protein
CT: computed tomography
CV: cardiovascular
CVD: cardiovascular disease
CVOT: cardiovascular outcome trial
DPP-4: dipeptidyl peptidase-4
eGFR: estimated glomerular filtration rate
eNOS: endothelial nitric oxide synthase
GIP: glucose-dependent insulinotropic polypeptide
GLP-1: glucagon-like peptide-1
GLP-1RA: glucagon-like peptide-1 receptor agonist
H₂FPEF: heavy, hypertensive, atrial fibrillation, pulmonary hypertension, elderly and filling pressure
HbA1c: glycated hemoglobin
HF: heart failure
HFA-PEFF: Heart Failure Association pre-test assessment, echocardiography and natriuretic peptide, functional testing and final aetiology
HFpEF: heart failure with preserved ejection fraction
HFrEF: heart failure with reduced ejection fraction
hs-CRP: high-sensitivity C-reactive protein
IL-6: interleukin-6
KCCQ: Kansas City Cardiomyopathy Questionnaire
LV: left ventricular
MACE: major adverse cardiovascular events
MI: myocardial infarction
NO: nitric oxide
NT-proBNP: N-terminal pro-B-type natriuretic peptide
PCI: percutaneous coronary intervention
PET: positron emission tomography
RCT: randomized controlled trial
SGLT2: sodium–glucose cotransporter 2
T2D: type 2 diabetes
TNF: tumor necrosis factor
UACR: urinary albumin-to-creatinine ratio
